# Finerenone Beyond Diabetic Kidney Disease: Emerging Evidence and Potential Systemic Implications

**DOI:** 10.3390/jcm15134852

**Published:** 2026-06-23

**Authors:** Mohanad Almaimani, Sadin Ayman Alamri

**Affiliations:** 1 Department of Internal Medicine, College of Medicine, University of Jeddah, Jeddah 23218, Saudi Arabia; 2 King Faisal Specialist Hospital and Research Center, Riyadh 11211, Saudi Arabia; sadeenalamrii@gmail.com

**Keywords:** finerenone, mineralocorticoid receptor, diabetic kidney disease, chronic kidney disease, heart failure

## Abstract

Mineralocorticoid receptor (MR) overactivation is a key driver of inflammation, fibrosis, and organ cross-talk across cardiorenal disease. Finerenone, a selective non-steroidal MR antagonist, has demonstrated robust renoprotective and cardioprotective benefits in patients with chronic kidney disease (CKD) and type 2 diabetes in large randomized clinical trials. Beyond its established role in diabetic kidney disease, emerging preclinical and clinical data suggest potential systemic effects through the attenuation of MR-driven inflammatory and fibrotic pathways. These include signals related to heart failure outcomes, atrial remodeling, pulmonary vascular biology, retinal microvascular integrity, and metabolic dysfunction. However, much of the evidence beyond established cardiorenal indications remains exploratory, based on preclinical studies, subgroup analyses, and post hoc evaluations. This review provides a critical synthesis of the established clinical evidence supporting finerenone in CKD and cardiovascular disease. It examines emerging, hypothesis-generating data regarding its potential systemic effects beyond diabetic kidney disease.

## 1. Introduction

Mineralocorticoid receptors (MRs) are ligand-activated transcription factors belonging to the nuclear receptor superfamily. Upon binding aldosterone, MRs regulate gene transcription involved in fluid and electrolyte homeostasis, blood pressure control, and vascular tone [1,2]. Beyond these classical roles, MR activation promotes inflammation, fibrosis, oxidative stress, and endothelial dysfunction. These maladaptive responses contribute to tissue injury in cardiovascular and renal systems, highlighting the dual physiological and pathological roles of aldosterone signaling [3].

**Figure 1:**
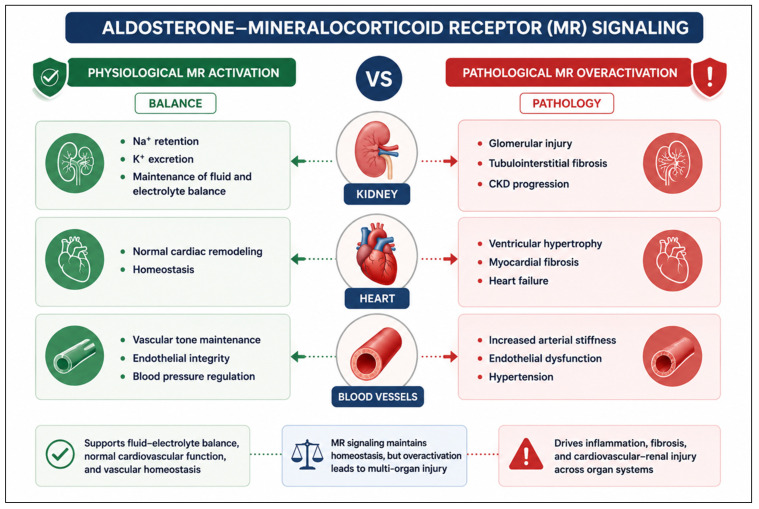
Aldosterone–mineralocorticoid receptor (MR) signaling: physiological versus pathological effects. Under physiological conditions, aldosterone-mediated MR activation maintains sodium and water homeostasis, potassium balance, vascular tone, and cardiac structure and function. In contrast, sustained or inappropriate MR overactivation promotes inflammation, fibrosis, and oxidative stress, leading to organ injury across the kidney, heart, and vasculature. In the kidney, this manifests as glomerular injury, tubulointerstitial fibrosis, and chronic kidney disease (CKD) progression. In the heart, MR overactivation contributes to ventricular hypertrophy, myocardial fibrosis, diastolic dysfunction, and heart failure (HF). In the vasculature, it leads to endothelial dysfunction, vascular inflammation, increased arterial stiffness, and hypertension. Abbreviations: CKD = chronic kidney disease; HF = heart failure; MR = mineralocorticoid receptor.

Although initially considered kidney-specific, MRs are widely expressed in extra-renal tissues, including the heart, vasculature, central nervous system (CNS), bone, lung, colon, adipose tissue, immune system, skin and retina [4]. Both aldosterone and cortisol bind to the MR with similar affinity; however, tissue-specific regulatory mechanisms determine ligand selectivity and downstream effects [5]. In epithelial tissues such as the distal nephron, 11β-hydroxysteroid dehydrogenase type 2 confers aldosterone specificity by inactivating cortisol. In contrast, non-epithelial tissues, such as the myocardium, lack such protection and are therefore more susceptible to cortisol-mediated MR overactivation [6]. This widespread distribution enables MR signaling to contribute to multi-organ injury beyond the kidney. The physiological and pathological effects of aldosterone-mediated MR activation across different organ systems are summarized in Figure 1, highlighting the central role of MR signaling in multi-organ injury.

Pathological MR overactivation has been implicated in experimental models of renal and cardiovascular injury [7,8]. Clinically, MR overactivation has been associated with insulin resistance and metabolic dysfunction in patients with hypertension, heart failure, and CKD. In the CNS, MR stimulation enhances sympathetic activity, whereas in vascular smooth muscle, it accelerates arterial stiffening and vascular aging [5].

These mechanistic insights have driven the development of more selective mineralocorticoid receptor antagonists to target MR-mediated injury while minimizing off-target effects. Despite treatment with renin–angiotensin–aldosterone system (RAAS) inhibitors, many patients with CKD or heart failure remain vulnerable to MR overactivation due to the aldosterone escape phenomenon [9]. This limitation provided the rationale for using mineralocorticoid receptor antagonists (MRAs). Steroidal MRAs, such as spironolactone and eplerenone, have demonstrated clear cardiovascular and renal benefits, but the risks of hyperkalemia and hormonal side effects have hampered their widespread use [10,11]. These limitations prompted the exploration of more selective and better-tolerated alternatives.

**Figure 2:**
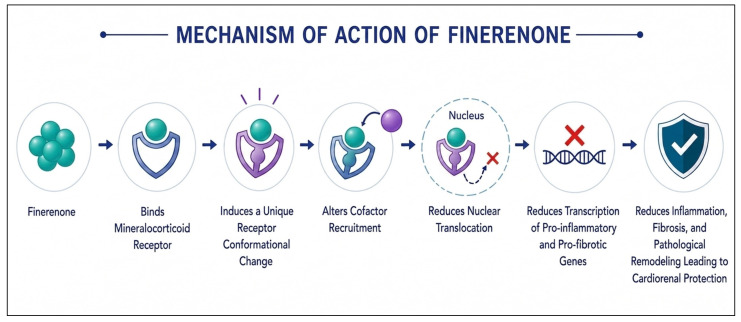
The mechanism of action of finerenone. Finerenone, a non-steroidal mineralocorticoid receptor antagonist, binds to the mineralocorticoid receptor and induces a unique receptor conformational change that alters cofactor recruitment and reduces nuclear translocation. These effects suppress the transcription of pro-inflammatory and pro-fibrotic genes, thereby attenuating inflammation, fibrosis, and pathological remodeling across multiple organ systems.

**Figure 3:**
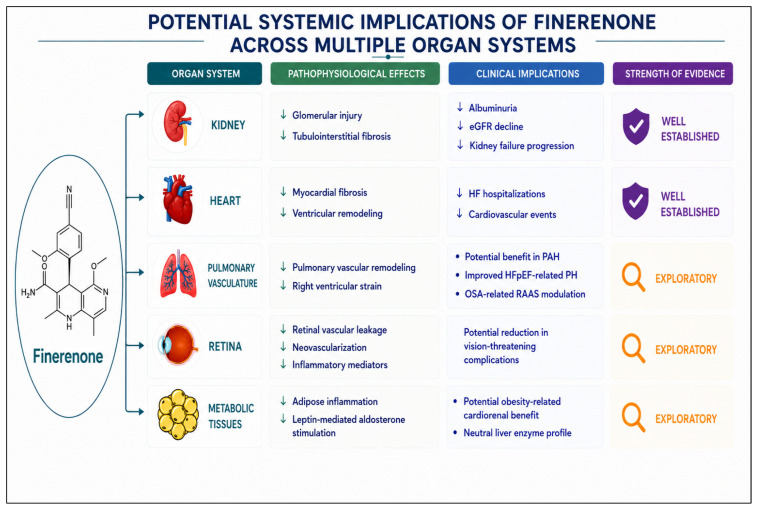
The potential systemic effects of finerenone across multiple organ systems. Finerenone modulates mineralocorticoid receptor–mediated inflammatory and fibrotic pathways. These effects may extend across multiple organ systems, including the kidney, heart, pulmonary vasculature, retina, and metabolic tissues. The strength of evidence varies across organ systems, with well-established clinical data supporting cardiorenal outcomes and more limited, exploratory evidence in other domains. Abbreviations: eGFR = estimated glomerular filtration rate; HFpEF = heart failure with preserved ejection fraction; OSA = obstructive sleep apnea; PAH = pulmonary arterial hypertension; RAAS = renin–angiotensin–aldosterone system.

Finerenone has emerged as a potent, highly selective, and non-steroidal MRA with unique pharmacological characteristics. Unlike spironolactone and eplerenone, finerenone exhibits greater receptor selectivity and minimal affinity for androgen and progesterone receptors, thereby reducing hormone-related adverse effects. Mechanistically, finerenone induces a unique conformational change in the mineralocorticoid receptor that alters receptor stability, cofactor recruitment, and nuclear translocation (Figure 2) [12]. From a pharmacokinetic perspective, finerenone has a relatively short half-life, lacks active metabolites, and exhibits limited central nervous system penetration due to its reduced lipophilicity, which may contribute to a lower risk of off-target effects [13]. Autoradiographic studies have revealed that finerenone is uniformly distributed between renal and cardiac tissues, compared to the renal-dominant distribution of steroidal MRAs [14]. Preclinical data have consistently demonstrated its ability to attenuate both renal and cardiac injuries [15]. The broad tissue distribution of MRs has generated interest in the potential systemic effects of finerenone beyond established cardiorenal indications, as summarized in Figure 3.

While the cardiorenal benefits of finerenone are supported by robust randomized clinical trial evidence, its potential effects across other organ systems remain less clearly defined. This review therefore examines both the established cardiorenal effects of finerenone and the emerging evidence supporting broader systemic applications, while highlighting the varying levels of evidence and the need for further investigation in areas where findings remain exploratory.

## 2. Methodology of Review

This narrative review was based on a targeted literature search of PubMed, Embase, and Google Scholar up to early 2026. Search terms included “finerenone,” “mineralocorticoid receptor antagonist,” “chronic kidney disease,” “diabetic kidney disease,” “heart failure,” “atrial fibrillation,” “pulmonary hypertension,” “diabetic retinopathy,” “obesity,” and “metabolic syndrome.” Priority was given to randomized controlled trials, major kidney and cardiovascular outcome studies, and high-quality meta-analyses where available. Preclinical studies, subgroup analyses, post hoc analyses, and observational studies were also included when relevant to emerging indications not yet supported by randomized evidence. Studies were selected based on their relevance to the scope of the review and their contribution to understanding the established and emerging effects of finerenone. No formal risk-of-bias assessment or predefined inclusion and exclusion criteria were applied, consistent with the narrative design of this review. Accordingly, findings from exploratory studies were interpreted cautiously and presented as hypothesis-generating where appropriate.

## 3. Renal Effects of Finerenone

### 3.1. Diabetic Kidney Disease (DKD)

The overactivation of MRs has been implicated in the pathogenesis of diabetic nephropathy, contributing to glomerular injury and tubulointerstitial fibrosis [15]. Finerenone exerts renoprotective effects through anti-inflammatory, anti-fibrotic, and anti-oxidative mechanisms [12,15]. This is evident in multiple preclinical studies. Yao et al. demonstrated that finerenone reduced albuminuria and renal fibrosis in a murine model of diabetic nephropathy, while also attenuating glomerulosclerosis and inflammatory signaling pathways [16]. Similarly, Lattenist et al. [17] showed that finerenone prevented CKD progression and ischemia–reperfusion injury in a Wistar rat model.

In clinical practice, renin–angiotensin system blockade remains the cornerstone of blood pressure control and kidney protection in patients with diabetic CKD. However, substantial residual cardiorenal risk persists, partly due to ongoing mineralocorticoid receptor overactivation, providing the rationale for adjunctive therapies such as finerenone [12].

In addition to basic research, the efficacy and safety of finerenone in DKD have also been evaluated in clinical trials. The ARTS-DN study, a randomized, double-blind, placebo-controlled Phase II trial, investigated patients with type 2 diabetes (T2D) and diabetic nephropathy who were administered a maximally tolerated dose of RAAS inhibitors. Finerenone reduced albuminuria in a dose-dependent manner with no significant adverse effects [18]. These results provide the foundation for subsequent phase III trials to evaluate the long-term renal and cardiovascular outcomes of finerenone.

Finerenone has been rigorously evaluated for its cardiorenal protective properties in large-scale phase-III clinical trials. In FIDELIO-DKD, which enrolled 5734 patients with type 2 diabetes and CKD, finerenone significantly reduced the composite kidney outcome of kidney failure, sustained ≥40% decline in eGFR, or renal death compared to placebo, along with fewer cardiovascular events, albeit with a higher incidence of hyperkalemia-related discontinuation (2.3% vs. 0.9%) [19].

The FIGARO-DKD trial, involving 7437 patients with earlier-stage CKD, confirmed a consistent cardiovascular benefit and trend toward renal protection, with similar safety findings [20]. The FIDELITY pooled analysis, combining both trials (*n* = 13,026), demonstrated that finerenone reduced kidney failure, sustained ≥57%, eGFR decline, or renal death by 23%, and lowered overall cardiovascular risk across the CKD spectrum, confirming its dual cardiorenal efficacy when added to optimized renin-angiotensin system blockade [21].

More recently, the FINE-ONE trial (NCT05901831) extended the evaluation of finerenone to patients with type 1 diabetes and CKD (*n* = 242; eGFR 25–<90 mL/min/1.73 m^2^; UACR 200–<5000 mg/g), demonstrating a significantly greater reduction in albuminuria compared with placebo over 6 months (approximately 25% relative reduction), with hyperkalemia occurring more frequently but leading to treatment discontinuation in only a small proportion of patients [22]. However, the trial was not designed to evaluate long-term kidney or cardiovascular outcomes; therefore, whether the observed reduction in albuminuria translates into durable cardiorenal benefit remains to be established.

Complementing these randomized data, real-world evidence from the prospective FINE-REAL study (NCT05348733) provides insight into the use of finerenone in routine clinical practice. In this interim analysis of approximately 2000 patients with a median follow-up of about 9 months, most patients were treated alongside contemporary background therapies. Finerenone was generally well tolerated, with a safety profile consistent with that observed in randomized trials; hyperkalemia occurred in approximately 8% of patients, leading to treatment discontinuation in about 1% [23].

Overall, the therapeutic landscape of DKD has evolved substantially with the availability of multiple disease-modifying therapies. Finerenone is now used alongside established treatments, including RAAS inhibitors and sodium–glucose cotransporter-2 inhibitors (SGLT2i), owing to their complementary mechanisms of action and cardiorenal benefits [24]. Supporting this strategy, the CONFIDENCE trial demonstrated greater reductions in albuminuria with combined finerenone and empagliflozin therapy compared with either agent alone, suggesting additive kidney protective effects [25].

### 3.2. Emerging Evidence in Non-Diabetic CKD

While the clinical evidence for finerenone is most robust in diabetic kidney disease, a substantial proportion of patients have CKD of non-diabetic etiology [26,27]. Although the underlying causes differ, the progression of many forms of CKD shares common pathophysiological pathways, including inflammation and fibrosis [6]. In this context, MR overactivation contributes to both hemodynamic and non-hemodynamic injury pathways, providing a mechanistic rationale for extending MR antagonism beyond diabetes [10].

This concept was evaluated in the phase III FIND-CKD trial (NCT05047263), which enrolled 1584 patients with non-diabetic CKD receiving optimized RAAS inhibitor therapy. Finerenone significantly slowed the decline in total eGFR slope over 32 months compared with placebo. Furthermore, as a key hierarchical secondary endpoint, finerenone reduced the risk of the composite kidney–cardiovascular outcome by 23% (HR 0.77, 95% CI 0.60–0.99) [28]. These findings provide the first phase III evidence supporting the use of finerenone in non-diabetic CKD.

## 4. Finerenone in Cardiovascular Disease

### 4.1. Heart Failure

The cardiovascular effects of finerenone are supported by evidence from randomized clinical trials and represent the most robust extension beyond renal outcomes. Aldosterone-mediated MR overactivation in cardiac tissues drives cardiomyocyte apoptosis and fibrosis, leading to myocardial hypertrophy, ventricular remodeling, arrhythmogenic effects, ischemic injury, and ultimately, heart failure [29,30]. Therefore, antagonizing this pathway is a cornerstone of heart failure therapy. This has been well proven historically with steroidal MRAs such as spironolactone and eplerenone in multiple large clinical trials, including RALES and EPHESUS, respectively [31,32]. These landmark trials established steroidal MRAs as essential components of guideline-directed medical therapy for heart failure with reduced ejection fraction (HFrEF). However, their use is often constrained in patients with CKD because of safety concerns, particularly the risk of hyperkalemia [33].

To address these limitations, finerenone was evaluated as a next-generation, non-steroidal MRA in the phase II ARTS-HF (Mineralocorticoid Receptor Antagonist Tolerability Study Heart Failure) trial. This study enrolled 1066 patients with worsening chronic HFrEF and concomitant T2D and/or CKD and compared finerenone with eplerenone at different doses. Finerenone achieved comparable reductions in NT-proBNP, defined as a ≥30% decrease from baseline, while demonstrating a more favorable renal and electrolyte safety profile [34].

The role of finerenone in heart failure management was subsequently reinforced in large phase III programs. Cardiovascular benefits were evident in the secondary outcomes of FIDELIO-DKD, primary outcomes of FIGARO-DKD, and the pooled FIDELITY analysis, as detailed in the DKD section [19,21]. More recently, the FINEARTS-HF trial, a double-blind, international trial, enrolled 6001 patients with heart failure and a left ventricular ejection fraction ≥ 40% to finerenone or placebo alongside standard therapy. Over a median follow-up period of 32 months, finerenone significantly reduced the composite endpoint of total worsening heart failure events and cardiovascular death, an effect primarily driven by fewer hospitalizations for heart failure [35]. These findings demonstrate that finerenone reduces heart failure morbidity in patients with mildly reduced (HFmrEF) or preserved ejection fraction (HFpEF), thereby expanding its therapeutic benefits beyond diabetic kidney disease.

Further support for the disease-modifying role of finerenone in heart failure comes from a recent systematic review and meta-analysis of over 21,000 patients with heart failure, T2D, or CKD, which demonstrated significant reductions in heart failure occurrence, progression, and hospitalization compared to placebo, along with an acceptable safety profile [36]. Consistent with FINEARTS-HF, these findings reinforce the position of finerenone as a promising therapeutic option in heart failure management.

While FINEARTS-HF demonstrated benefits in HFmrEF and HFpEF, the full potential of finerenone in heart failure, particularly its impact across the entire ejection fraction spectrum and in direct comparison with established steroidal MRAs, requires further investigation. Several ongoing trials have directly addressed these gaps. FINALITY-HF (NCT06033950) is actively recruiting patients with HFrEF who are intolerant or ineligible for steroidal MRAs, whereas REDEFINE-HF (NCT06008197) evaluates finerenone in hospitalized patients with HFmrEF/HFpEF. These studies will yield critical insights into the comparative efficacy and safety of finerenone across diverse heart failure populations and help shape its future role in treatment algorithms.

### 4.2. Atrial Tachyarrhythmia

While most large trials have focused on ventricular function and heart failure outcomes, MR overactivation is also a key driver of atrial remodeling and arrhythmogenesis. Preclinical studies by Reil et al. (2012) and Lavall et al. (2014) demonstrated that aldosterone-induced MR activation promotes atrial fibrosis, dilation, and conduction heterogeneity, pathological substrates that predispose patients to atrial fibrillation [37,38]. Building on this foundation, later experimental work by Lavall et al. (2019) showed that finerenone effectively prevented MR-mediated left-atrial fibrosis and dilation in experimental rat models, underscoring its direct anti-fibrotic action in atrial tissue [39].

Clinically, these mechanistic insights have been substantiated by major outcome trials. A pre-specified secondary analysis of the FIDELIO-DKD trial revealed a significantly lower incidence of new-onset atrial fibrillation (AF) or atrial flutter (AFL) among patients receiving finerenone compared with placebo, with consistent cardiorenal benefits regardless of AF/AFL at baseline [40]. More recently, the FINE-HEART pooled analysis, integrating data from FIDELIO-DKD, FIGARO-DKD, and FINEARTS-HF, demonstrated a lower incidence of AF/AFL among patients receiving finerenone across the cardiorenal spectrum [41]. Together, these findings suggest that finerenone may influence atrial remodeling and susceptibility to atrial tachyarrhythmias; however, dedicated prospective studies are required to determine whether these observations translate into clinically meaningful antiarrhythmic benefits. The key characteristics and outcomes of major clinical trials evaluating finerenone across the cardiorenal spectrum are summarized in Table 1.

## 5. Emerging Systemic Effects Beyond the Established Cardiorenal Indications

### 5.1. Pulmonary Vascular and Respiratory Effects

Evidence regarding pulmonary effects is currently limited to preclinical studies and exploratory clinical observations. Beyond its cardiovascular implications, MR overactivation has been implicated in pulmonary vascular diseases. Elevated aldosterone levels, demonstrated in both human and experimental models of pulmonary arterial hypertension (PAH), activate MR signaling in endothelial and vascular smooth muscle cells, promoting oxidative stress, inflammation, and pulmonary vascular remodeling [43]. In preclinical PAH models, finerenone markedly attenuated these structural changes, reducing smooth muscle proliferation and improving pulmonary hemodynamic parameters, including mean pulmonary arterial pressure (mPAP), right ventricular systolic pressure (RVSP), and right ventricular hypertrophy [44]. Mechanistic studies have further revealed that MR activation in pulmonary endothelial and smooth muscle cells drives distinct pro-fibrotic and vasoconstrictive pathways, underscoring the importance of cell-specific MR modulation in disease progression [45,46]. Moreover, MR overactivation contributes to pulmonary hypertension secondary to left-sided heart disease, particularly heart failure with preserved ejection fraction (HFpEF), which exacerbates right ventricular dysfunction and vascular stiffness [47]. Collectively, these findings support a potential role for MR signaling in pulmonary vascular pathology and provide a mechanistic rationale for further investigation of finerenone in pulmonary vascular disease.

In addition to vascular pathology, aldosterone excess also affects pulmonary function through mechanisms related to sleep-disordered breathing. Patients with obstructive sleep apnea (OSA), particularly those with resistant hypertension, exhibit elevated aldosterone levels that exacerbate upper airway obstruction through nocturnal rostral fluid shifts and elevated blood pressure [48]. In turn, chronic intermittent hypoxia and sympathetic activation further stimulate aldosterone secretion by upregulating the RAAS, perpetuating this vicious cycle [49]. MR antagonism has been associated with improvements in OSA severity and blood pressure control in patients with resistant hypertension [50,51]. Whether these observations translate into a clinically meaningful role for finerenone in OSA-related hypertension remains unknown and warrants dedicated investigation.

In addition to OSA, chronic obstructive pulmonary disease (COPD) is another pulmonary comorbidity that frequently accompanies heart failure and is linked to adverse outcomes. In the prespecified COPD analysis of the FINEARTS-HF trial, patients with HFmrEF or HFpEF and concomitant COPD exhibited a greater symptom burden, elevated biomarkers, and worse functional status than those without COPD. Finerenone demonstrated consistent reductions in cardiovascular death and heart failure events, irrespective of COPD status, and improved the patient-reported quality of life [52]. However, these findings should be interpreted cautiously, as the analysis was exploratory and does not establish a specific therapeutic role for finerenone in COPD.

### 5.2. Retinal Effects

Available data on retinal effects are primarily derived from preclinical models and exploratory clinical analyses and should be considered hypothesis-generating. The retina possesses a local RAAS, through which MR signaling directly influences vascular integrity and homeostasis. Excess MR activation promotes endothelial dysfunction, oxidative stress, inflammatory cytokine expression, and leukocyte adhesion within retinal vessels, driving the vascular injury and neovascularization observed in diabetic and ischemic retinopathy models [53,54].

Preclinical studies have consistently demonstrated favorable effects of the MR blockade in this setting. Wilkinson-Berka et al. first identified a functional retinal aldosterone system showing local expression of aldosterone synthase and MR, and demonstrated that pharmacological MR antagonism significantly reduced retinal vascular injury and leukocyte adhesion in oxygen-induced retinopathy (OIR) rat models [53]. Subsequently, Jerome et al. [54] evaluated finerenone in two experimental models: transgenic diabetic–hypertensive rats and OIR. In the former, finerenone reduced retinal vascular leakage and downregulated pro-angiogenic and inflammatory mediators, whereas in the latter, it markedly limited pathological neovascularization and enhanced retinal regulatory T-cell activity, indicating that its protective actions extend beyond vascular stabilization to include an immunomodulatory component.

These experimental findings provide a rationale for investigating whether finerenone may delay the progression of diabetic retinopathy (DR). To address this, a pooled analysis of routine ophthalmologic examinations from participants in the FIDELIO-DKD and FIGARO-DKD trials was conducted in two studies—ReFineDR and DeFineDR- to assess the effect of finerenone on the development of vision-threatening complications (VTCs), defined as progression to proliferative DR, clinically significant macular edema, or the need for ocular intervention. Over approximately two years of follow-up, finerenone was associated with a delayed onset of VTCs compared with placebo, with consistent findings irrespective of baseline HbA1c [55]. These findings provide preliminary clinical support for the hypothesis that finerenone may influence retinal microvascular disease; however, dedicated ophthalmologic outcome trials are required to confirm these observations.

Further insights are anticipated from the ongoing FINE-REAL study, a prospective, multicenter observational investigation assessing the real-world use, effectiveness, and safety of finerenone in patients with CKD and T2D [56]. Among its secondary objectives is the evaluation of new-onset diabetic retinopathy or the progression of pre-existing disease, providing complementary evidence to findings from controlled trials. The outcomes of this study may further clarify the potential role of finerenone in diabetic retinopathy.

### 5.3. Metabolic and Endocrine Effects

Evidence for metabolic and endocrine effects remains limited and heterogeneous, largely based on preclinical studies and subgroup analyses. Obesity is a major public health challenge associated with multiple comorbidities, including T2D, hypertension, and CKD [57]. Neurohormonal dysregulation significantly contributes to organ damage in obesity, with clinical and mechanistic studies demonstrating an association between circulating aldosterone levels and body mass and adiposity [58,59]. Adipose tissue contributes to RAAS activation and promotes leptin-mediated stimulation of adrenal aldosterone secretion. MRs are also expressed in adipose tissue [60]. Excessive MR activation in obese individuals leads to deleterious systemic effects, including vascular inflammation, fibrosis, and endothelial dysfunction [2].

The therapeutic relevance of selective MR antagonism in obesity has been demonstrated in both experimental and clinical studies on finerenone. In preclinical models of obese mice, finerenone improved left ventricular compliance and facilitated cardiovascular recovery following normalization of food intake after a high-fat diet, indicating reversal of obesity-related myocardial dysfunction [61]. Clinically, a retrospective study of patients with obesity-related glomerulopathy showed that finerenone was associated with reductions in proteinuria and blood pressure while stabilizing the estimated glomerular filtration rate over a one-year follow-up period [62]. In larger cardiovascular populations, a prespecified analysis of the FINEARTS-HF trial (*n* = 6001) demonstrated that finerenone’s benefits on clinical outcomes and symptoms of HFmrEF or HFpEF were consistent across BMI categories, with a signal toward greater efficacy in individuals with higher BMI [63]. Collectively, these findings suggest that finerenone may have therapeutic relevance in obesity-associated cardiorenal disease, although dedicated studies in obesity-specific populations remain limited.

A post hoc analysis of the FIDELITY dataset evaluated the effects of finerenone on liver enzymes and kidney and cardiovascular outcomes in patients with CKD and T2D, stratified according to surrogate markers of hepatic steatosis, inflammation, and fibrosis, including the hepatic steatosis index, liver enzymes, and FIB-4 scores [64]. Liver enzyme levels remained stable and comparable between the finerenone and placebo groups, indicating a neutral effect on the liver enzyme parameters. Finerenone consistently reduced the risk of composite kidney outcomes irrespective of baseline liver abnormalities. Higher FIB-4 scores were associated with increased cardiovascular event rates, and finerenone was associated with a 24–52% relative risk reduction in composite cardiovascular outcomes across fibrosis subgroups, with a greater benefit observed in patients with more advanced fibrosis [64].

Within the framework of metabolic syndrome, where obesity, insulin resistance, and non-alcoholic fatty liver disease are closely interconnected, these findings suggest that finerenone retains its cardiorenal protective effects despite a neutral impact on hepatic injury markers. Although NAFLD is increasingly recognized as part of a broader cardiometabolic disease spectrum, the direct effects of finerenone on liver disease remain uncertain [65].

## 6. Limitations

Despite the growing evidence supporting the use of finerenone in DKD and heart failure, several limitations of the current evidence base should be acknowledged. First, although finerenone is generally associated with a lower incidence of hyperkalemia than steroidal MRAs, hyperkalemia remains an important adverse effect, particularly in patients with advanced CKD and those receiving concomitant RAAS inhibitors. Careful patient selection, including assessment of baseline kidney function and serum potassium levels, together with regular potassium monitoring and appropriate dose adjustments, remains essential in clinical practice.

Second, direct head-to-head comparisons between finerenone and established steroidal MRAs, such as spironolactone and eplerenone, are currently lacking. Consequently, the relative efficacy, safety, and cost-effectiveness of these agents across different cardiorenal populations remain incompletely defined. In addition, while available clinical trials have demonstrated favorable safety and efficacy profiles, long-term data extending beyond the duration of current studies remain limited.

Third, the expanding evidence for finerenone beyond established cardiorenal indications should be interpreted cautiously. While emerging data in pulmonary vascular disease, retinal disorders, and metabolic dysfunction are biologically plausible and supported by preclinical studies and exploratory clinical analyses, these findings remain largely hypothesis-generating and require confirmation in dedicated prospective trials before routine clinical application can be considered.

Finally, issues related to drug cost, accessibility, and healthcare system adoption may influence the real-world implementation of finerenone and should be considered when translating trial findings into clinical practice.

## 7. Future Directions

The therapeutic landscape of finerenone continues to evolve beyond its established role in DKD and heart failure. Recent evidence from FIND-CKD has extended the benefits of finerenone to patients with non-diabetic CKD, while FINE-ONE has expanded its investigation into T1D-associated CKD, although evidence for hard renal and cardiovascular endpoints remains limited [22,28]. In parallel, combination therapy has emerged as an important area of investigation. Given their complementary mechanisms of action, finerenone and SGLT2i may provide additive cardiorenal protection, as demonstrated by the greater reduction in albuminuria observed with combined finerenone and empagliflozin therapy in the CONFIDENCE trial [25].

Additional studies will further define the role of finerenone across the cardiorenal spectrum. Ongoing trials such as FINALITY-HF and REDEFINE-HF are expected to clarify its efficacy and safety in broader heart failure populations and help establish its position relative to established steroidal MRAs. Beyond cardiorenal indications, dedicated prospective studies are needed to determine whether the promising pulmonary, retinal, and metabolic signals observed in preclinical and exploratory clinical analyses translate into clinically meaningful therapeutic benefits. Collectively, these investigations will help define the future role of finerenone within precision cardiorenal and metabolic medicine.

## 8. Conclusions

Finerenone represents an important advance in MR antagonism, with robust evidence supporting its cardiorenal benefits in patients with CKD and T2D, as well as in selected heart failure populations. By targeting inflammatory and fibrotic pathways implicated in disease progression, finerenone provides therapeutic benefits that extend beyond traditional hemodynamic mechanisms.

Emerging evidence has generated interest in the potential role of finerenone across additional organ systems, including the atria, pulmonary vasculature, retina, and metabolic tissues. However, the strength of evidence varies considerably across these domains, and many findings remain exploratory or hypothesis-generating. Ongoing and future studies will further define the role of finerenone across the broader cardiorenal spectrum and determine whether these emerging signals translate into clinically meaningful therapeutic applications.

## Figures and Tables

**Table 1:** Major clinical trials evaluating finerenone across cardiorenal spectrum.

Study (Year)	Population	Outcome/Endpoint	Key Findings
**Early-Phase Trials**			
ARTS [42] (2013)	HFrEF (LVEF ≤ 40%) with CKD (eGFR 30–60 mL/min/1.73 m^2^)	Safety and cardiorenal biomarkers	Comparable reductions in BNP/NT-proBNP and albuminuria versus spironolactone, with less hyperkalemia (5.3% vs. 12.7%).
ARTS-DN [18] (2015)	T2D with albuminuric CKD (eGFR > 30 mL/min/1.73 m^2^; UACR ≥30 mg/g)	Change in UACR at Day 90	Dose-dependent reduction in albuminuria (up to 38%), with low hyperkalemia rates (1.7–3.2%).
ARTS-HF [34] (2016)	Worsening HFrEF (LVEF ≤ 40%) with CKD and/or T2D	≥30% reduction in NT-proBNP at Day 90	Non-inferior NT-proBNP reduction versus eplerenone, with low hyperkalemia incidence (4.3%).
**Pivotal DKD Trials**			
FIDELIO-DKD [19] (2020)	T2D and CKD (eGFR 25–75 mL/min/1.73 m^2^; UACR 30–5000 mg/g)	Kidney composite outcome	Reduced kidney disease progression (HR 0.82); increased hyperkalemia-related discontinuation (2.3% vs. 0.9%).
FIGARO-DKD [20] (2021)	T2D and CKD (eGFR >25–90 mL/min/1.73 m^2^; UACR 30–5000 mg/g)	CV composite outcome	Reduced cardiovascular events (HR 0.87); increased hyperkalemia incidence (10.8% vs. 5.3%).
FIDELITY [21] (2022)	Pooled analysis of FIDELIO-DKD and FIGARO-DKD	Cardiorenal composite outcomes	Reduced cardiovascular events (HR 0.86) and kidney outcomes (HR 0.77); increased hyperkalemia-related discontinuation (1.7% vs. 0.6%).
**Non-Diabetic CKD Trials**			
FIND-CKD [28] (2026)	Non-diabetic CKD (eGFR 25–<90 mL/min/1.73 m^2^; UACR 200–≤3500 mg/g)	Total eGFR slope	Slowed total eGFR decline over 32 months; reduced the hierarchical composite cardiorenal outcome by 23% (HR 0.77). Increased hyperkalemia incidence (17.0% vs. 13.3%).
**Heart Failure Trials**			
FINEARTS-HF [35] (2024)	HFmrEF/HFpEF (LVEF ≥ 40%; NYHA class II–IV)	CV death and total HF events	Reduced total HF events and cardiovascular death (HR 0.84), with consistent benefit across HF subgroups.

Abbreviations: BNP = B-type natriuretic peptide; NT-proBNP = N-terminal pro-B-type natriuretic peptide; CKD = chronic kidney disease; CV = cardiovascular; eGFR = estimated glomerular filtration rate; HF = heart failure; HFrEF = heart failure with reduced ejection fraction; HFmrEF = heart failure with mildly reduced ejection fraction; HFpEF = heart failure with preserved ejection fraction; LVEF = left ventricular ejection fraction; NYHA = New York Heart Association; T2D = type 2 diabetes; UACR = urine albumin-to-creatinine ratio.

## Data Availability

No new data were created or analyzed in this study.

## Abbreviations

The following abbreviations are used in this manuscript:

AF/AFL	Atrial fibrillation/flutter
ATP	Adenosine triphosphate
BMI	Body mass index
BNP	B-type natriuretic peptide
CKD	Chronic kidney disease
CNS	Central nervous system
COPD	Chronic obstructive pulmonary disease
CV	Cardiovascular
DKD	Diabetic kidney disease
DR	Diabetic retinopathy
eGFR	Estimated glomerular filtration rate
FIB-4	Fibrosis-4 index
HbA1c	Glycated hemoglobin
HF	Heart failure
HFmrEF	Heart failure with mildly reduced ejection fraction
HFpEF	Heart failure with preserved ejection fraction
HFrEF	Heart failure with reduced ejection fraction
MR	Mineralocorticoid receptor
MRA	Mineralocorticoid receptor antagonist
mPAP	Mean pulmonary arterial pressure
NT-proBNP	N-terminal pro–B-type natriuretic peptide
NYHA	New York Heart Association
OIR	Oxygen-induced retinopathy
OSA	Obstructive sleep apnea
PAH	Pulmonary arterial hypertension
RAAS	Renin–angiotensin–aldosterone system
RVSP	Right ventricular systolic pressure
T2D	Type 2 diabetes
UACR	Urinary albumin-to-creatinine ratio
VTC	Vision-threatening complication
